# Nurse leaders' and digital service developers' perceptions of the future role of artificial intelligence in specialized medical care: An interview study

**DOI:** 10.1111/jonm.13769

**Published:** 2022-08-21

**Authors:** Elina Laukka, Mira Hammarén, Outi Kanste

**Affiliations:** ^1^ Research Unit of Nursing Science and Health Management University of Oulu Oulu Finland; ^2^ Medical Research Center Oulu University Hospital Oulu Finland

**Keywords:** artificial intelligence, content analysis, digitalization, interview, nurse manager, specialized medical care

## Abstract

**Aim:**

To describe nurse leaders' and digital service developers' perceptions of the future role of artificial intelligence (AI) in specialized medical care.

**Background:**

Use of AI has rapidly increased in health care. However, nurse leaders' and developers' perceptions of AI and its future in specialized medical care remain under‐researched.

**Method:**

Descriptive qualitative methodology was applied. Data were collected through six focus groups, and interviews with nurse leaders (*n* = 20) and digital service developers (*n* = 10) conducted remotely in 2021 at a university hospital in Finland. The data were subjected to inductive content analysis.

**Results:**

The data yielded 25 sub‐categories, 10 categories and three main categories of participants' perceptions. The main categories were designated AI transforming: work, care and services and organizations.

**Conclusions:**

According to our respondents, AI will have a significant future role in specialized medical care, but it will likely reinforce, rather than replace, clinicians or traditional care. They also believe that it may have several positive consequences for clinicians' and leaders' work as well as for organizations and patients.

**Implications for nursing management:**

Nurse leaders should be familiar with the potential of AI, but also aware of risks. Such leaders may provide betters support for development of AI‐based health services that improve clinicians' workflows.

## 1 INTRODUCTION

Artificial intelligence (AI) is a term coined by McCarthy (1956) who defined it as ‘the science and engineering of making intelligent machines, especially intelligent computer programs’. Strictly, this refers to the discipline of AI rather than AI per se, which generally refers to a collection of technologies that mimic (or surpass) key human intellectual functions (Davenport & Kalakota, 2019). It has been seen as an answer to some of the challenges confronting today's health care sector. For example, AI‐based solutions for checking COVID‐19 symptoms have been developed (Scott & Coiera, 2020). Additionally, due to rapid digitalization of health care, an enormous and continuously expanding volume of patient data can only be dealt with by AI‐based processing. Thus, AI already seems to play indispensable roles in our health care systems, and its significance will surely increase.

Inter alia, AI systems already have excellent pattern recognition capacities (Kim et al., 2019). Such abilities are rooted in ‘machine learning’ programmes that process input data by applying algorithms that iteratively seek patterns, check matches of their outputs to test datasets and refine their parameters, thereby validating the system (Haenlein & Kaplan, 2019). Most AI technologies, such as machine learning and deep learning, already have immediate relevance in health care (Davenport & Kalakota, 2019; Kim et al., 2019). From a health care perspective, AI brings a ‘paradigm shift to healthcare, powered by increasing availability of healthcare data and rapid processes of analytic techniques’ (Jiang et al., 2017).

AI has already been used to enhance clinical decision‐making (Diprose et al., 2020) and has been seen as potentially transformative (Sarwar et al., 2019). In addition, there are increasing numbers of patient engagement and adherence applications, and AI has already been applied in some administrative processes (Davenport & Kalakota, 2019). However, its implementation raises complex ethical, legal, clinical and safety issues because of a lack of understanding of how AI models generate their outputs (Scott et al., 2021)—commonly known as ‘the black box problem’ (Neri et al., 2020).

AI technologies are changing the nursing profession (Ronquillo et al., 2021), and nurse leaders need to be visionaries (Ahonen et al., 2016) who deeply consider the future of nursing, including the implications of AI. However, various health care professionals, particularly clinicians, reportedly have mixed attitudes towards AI (Abdullah & Fakieh, 2020), and it has been claimed that they understand neither how AI uses algorithms nor the inner workings of algorithms (Romero‐Brufau et al., 2020). This lack of knowledge may increase anxiety and arouse conflicting emotions in clinical staff (Abdullah & Fakieh, 2020), which may affect their perceptions of AI.

The ability of leaders to envision a better future strengthens the possibility of successfully addressing the challenges confronting health care (Malloch & Melnyk, 2013). Hence, nurse leaders need to foster positive attitudes towards AI technologies (Ronquillo et al., 2021), and to assist AI's beneficial deployment in future health care there are clear needs to understand nurse leaders' and developers' current perceptions of AI. This would provide insights into how AI could be developed to best serve health care organizations, clinicians' workflows and patient care. Such insights would be highly valuable for developers of AI, health care organizations and decision‐makers seeking to develop and implement effective AI‐based solutions. Therefore, the aim of the study presented here was to elicit nurse leaders' and digital service developers' perceptions of the future role of AI in specialized medical care. The specific research question posed was, What kind of perceptions do nurse leaders and digital service developers have of the future role of AI?

## 2 METHODS

### 2.1 Design and setting

**FIGURE 1:**
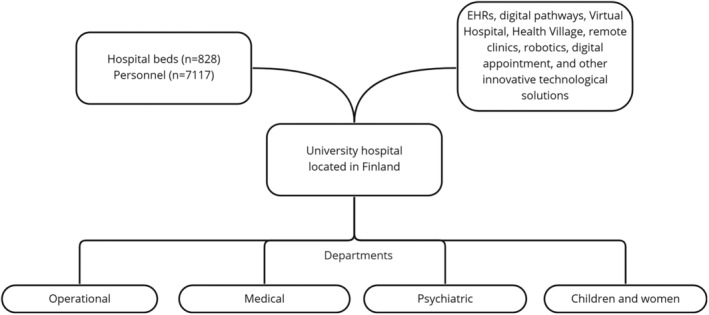
Description of university hospital (research organization)

We designed a descriptive qualitative study to describe Finnish nurse leaders' and digital service developers' perspectives on the future role of AI in specialized medical care (Polit & Beck, 2017). A qualitative design was chosen since it is suitable for exploring phenomena that are poorly and incompletely understood (Polit & Beck, 2017). The interviews were conducted in a Finnish university hospital with 828 hospital beds and 7117 personnel (Figure 1). We focused on staff working in specialized medical care units at this hospital partly because it is a pioneer in the development and implementation of digital health services, such as digital pathways, Virtual Hospital systems, digital Health Village platforms, remote clinics, robotics and other innovative technological solutions. During all relevant stages of the study, we followed the Consolidated Criteria for Reporting Qualitative Research (COREQ) (Tong et al., 2007).

### 2.2 Participants and recruitment

The participants were frontline nurse leaders (head nurses and assistant head nurses), middle managers (*n* = 20) and digital service developers (*n* = 10) working in specialized medical care in operational, medical, psychiatric, children and women departments and group services at the university hospital. The digital service developers were professionals in either health care or information technology and worked full‐time or part‐time on tasks or projects related to the development of digital services in specialized medical care. Both the nurse leaders and digital service developers were employed by the hospital. Eligible interviewees had to have experience of digital health services (e.g., electronic health records and/or patient portals), and understand current roles of digital services and digitalization in the organization's operations and strategy.

We used two recruiting strategies, depending on how hospital departments wished us to recruit interviewees. Initially, purposive sampling was used to acquire rich data (Polit & Beck, 2017). Two out of four departments gave us a list of five persons to contact concerning the focus groups. Two other departments gave us a list of nursing directors, head nurses and assistant head nurses. We sent emails to all of the named staff (*n* = 140) and recruited 22 interviewees. However, only 20 participated in group interviews due to scheduling difficulties. The nursing director gave us five names of digital service developers to contact, then a snowball sampling approach was employed to recruit digital service developers (*n* = 10) (Polit & Beck, 2017).

### 2.3 Data collection and materials

A semi‐structured interview guide, covering four common themes (competence, leadership, work wellbeing and AI), was developed by the research team, including three experienced qualitative researchers, and content experts on nursing, digitization and leadership in health care. Use of the semi‐structured interview guide fostered the emergence of relevant content from participants (Polit & Beck, 2017). Questions on the AI theme were based on earlier research (Abdullah & Fakieh, 2020; Yu et al., 2018) and included one on the future role of AI in specialized medical care.

The guide (Table 1) was pretested in a focus group with four leaders and individual interviews with two digital service developers. We asked these participants to evaluate the understandability and relevance of the interview guide, and we subjectively assessed the suitability of the duration of the sessions, group size, and group dynamics. The interviewees stated that the questions were understandable.

**TABLE 1:** The semi‐structured interview guide

Interview questions
What do you think about the role of AI in health care in the future?
How do you think AI will affect now and in the future?
Assisting questions:
Work in health care: Including your own work, and work of professionals generally?
Services: Patient and customer work?
Organizations: In terms of productivity, effectiveness, quality, safety and human or patient‐centeredness?

Data were collected by two researchers (EL, AY) from the focus groups and one researcher (MH, AY) from the individual interviews between July and November 2021 using Microsoft Teams. Six focus groups interviews were conducted, in each cause with three or four group members (Table 2). According to relevant methodological literature, numbers of both the interviewees and focus groups were appropriate (Tong et al., 2007). The groups were formed so that frontline leaders and middle managers were interviewed separately. Background information on the participants was collected by a Webropol survey (Table 3). Saturating data were obtained from both the focus groups and individual interviews (Kyngäs et al., 2020).

**TABLE 2:** Description of the focus groups

Group	Number of participants	Work task (current)	Age (years) mean (range)	Work experience (years) in leader position mean (range)
G1	4	Head nurses	51.5 (44.0–61.0)	11.0 (6.0–21.2)
G2	4	Head nurses	49.0 (44.0–55.0)	9.5 (5.0–14.0)
G3	3	Two assistant head nurses and one head nurse	50.3 (43.0–55.0)	10.0 (7.1–15.0)
G4	3	Assistant head nurses	46.0 (39.0–57.0)	6.3 (1.8–12.0)
G5	3	Assistant head nurses	52.0 (43.0–60.0)	4.9 (3.8–7.0)
G6	3	Nurse middle managers	59.0 (56.0–61.0)	19.2 (16.6–22.0)

**TABLE 3:** Nurse leaders' and digital service developers' demographics

Variable	Leaders	Developers
	(*n* = 20)	(*n* = 10)
	*F*	*F*
Gender		
Female	20	8
Male	–	2
Educational level		
Master's university degree or higher	11	4
Bachelor's degree or lower	9	6
Field of education		
Nursing/health care	20	7
Information technology	–	3
Work task (current)		
Head nurse	9	–
Assistant head nurse	8	–
Nurse middle manager	3	–
Project/expert work	–	6
Planner/designer	–	4
	Mean	Mean
Age (years)	51.2^a^	42.4^b^
Work experience (years)		
In current work^b^	6.3^c^	2.9^d^
In leader position in health care^c^	10.2^e^	
In health care^d^		18.9^f^

^a^ SD: 7.1; min–max: 39.0–61.0.

^b^ SD: 5.6; min–max: 34.0–49.0.

^c^ SD: 4.5; min–max: 0.8–21.2.

^d^ SD: 2.5; min–max: 0.6–8.2.

^e^ SD: 6.0; min–max: 1.8–22.0.

^f^ SD: 5.3; min–max: 9.0–25.0.

With the participants' permission, the interviews were audio‐recorded using Microsoft Teams then transcribed verbatim, with tape recording as a back‐up. The duration of the focus group interviews ranged from 73 to 106 min, and individual interviews from 36 to 72 min (total duration 1011 min). The transcripts comprised 333 pages of 12‐point Calibri (body) font with 1.15 line spacing.

### 2.4 Ethical considerations

No formal ethical approval was required according to the hospital's Research Ethics Committee, since the interviews did not concern patients, minors or anyone with any category of physical or mental exemption from the ability to provide informed consent (World Medical Association Declaration of Helsinki, 2013). However, research permission was granted, and the participating organization considered ethical aspects of the study. Before the interviews, participants received written and spoken information concerning the study and researchers, and data protection notification. Written consent was then obtained from each participant electronically.

### 2.5 Data analysis

The data were subjected to inductive content analysis, because it provides a systematic and objective means of describing poorly understood phenomena (Kyngäs et al., 2020). For this, NVivo software version 1.5 (QSR International Pty Ltd) was used. In addition to the transcripts, demographic data were obtained and are reported using descriptive statistics.

Two researchers (EL, MH) initially conducted the data analysis, and after forming the main categories they conferred with the third researcher (OK). All phases of content analysis were first done independently, and after each phase, the two researchers discussed the analytical process. The two researchers first read the data repeatedly, seeking insights. Secondly, they coded it using words or statements that related to the same central meaning as analysis units (Kyngäs et al., 2020). The original expressions were reduced to convert them into codes (*n* = 136). Thirdly, the codes were grouped into sub‐categories based on similarities/differences, then the sub‐categories were named according to their content. The categories were created through interpretation by identifying associated sub‐categories. The main categories were formed by combining categories. Finally, the third researcher was consulted, the researchers reached consensus regarding the categories' content, and assigned names to the categories.

### 2.6 Rigour

The trustworthiness of qualitative research depends on its credibility, dependability, confirmability, authenticity and transferability (Kyngäs et al., 2020; Lincoln & Guba, 1985). We sought to maximize the credibility of our work, within time and resource constraints, by selecting appropriate interviewees and ensuring that the data were saturated. The inductive content analysis procedure described above, involving two researchers initially conducting the independently then the third researcher commenting on the categories and comparing them to original data, was designed to strengthen dependability. To improve confirmability, the third researcher checked, as far as possible, that the findings were solely shaped by the collected data rather than researcher bias. To enable readers to judge the findings' transferability, we have provided relevant contextual information about the study setting and participants. In efforts to ensure authenticity we have included citations illustrating the connection between the findings and data (Kyngäs et al., 2020).

## 3 RESULTS

**FIGURE 2:**
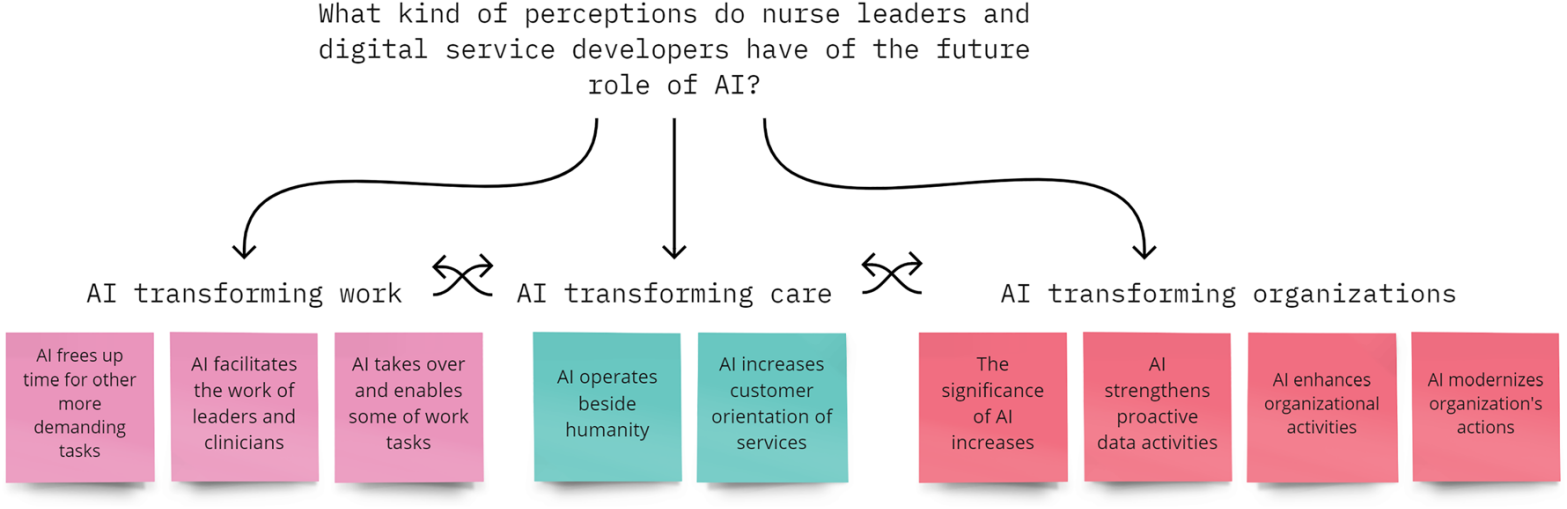
Nurse leaders' and digital service developers' perceptions of the future role of artificial intelligence (AI)

Analysis of the data revealed three main categories of content regarding nurse leaders' and digital service developers' perceptions of the future role of AI in specialized medical care. These are AI transforming: (1) work, (2) care and services and (3) organizations (Figure 2). These main categories included 10 categories, which included 25 sub‐categories (Table 4).

**TABLE 4:** Sub‐categories, categories and main categories created using inductive content analysis

Sub‐category (*n* = 25)	Category (*n* = 10)	Main category (*n* = 3)
AI can enhance clinicians' interactions with patients	AI frees up time for other more demanding tasks	AI transforming work
AI frees up clinicians' time		
AI supports leaders and clinicians	AI facilitates the work of leaders and clinicians	
AI makes clinicians' work easier		
AI can distribute medicines	AI takes over and enables some work tasks	
AI takes over routine work		
AI replaces some work tasks		
AI increases the need for IT clinicians		
AI transforms and enables tasks		
AI will not replace interaction	AI operates beside humanity	AI transforming care and services
AI will not replace all traditional services		
AI will not replace humans in future		
AI improves patient safety	AI increases customer orientation of services	
AI increases patients' active role in their care		
AI enhances personalized care		
AI enhances services and their availability		
AI's role will increase in future	AI's significance will increase	AI transforming organizations
AI will have a significant and major role in the future		
AI enables more efficient data management	AI strengthens proactive data management	
AI foresees the future		
AI saves resources	AI enhances organizational activities	
AI increases productivity		
AI increases efficiency		
AI changes organization's image	AI modernizes organization's actions	
AI shapes interactions in an organization		

### 3.1 Transforming work

The participating nurse leaders and developers expressed beliefs that AI will transform work in specialized medical care, free up clinicians' time and thus enable more interaction with patients. They also suggested that AI would perform more patient recording and routine work, leaving clinicians more time to focus on other demanding tasks currently beyond AI.

‘Patients can interact with AI on specific issues and seek advice leaving nurses time to perform other tasks …’ Assistant head nurse/Group 5

Interviewees also expressed perceptions that AI could support leaders and clinicians in their work, inter alia by providing management tools for leaders. According to interviewees, AI will become a co‐worker, performing work tasks, advising, anticipating actions and enhancing work by reducing human error and complementing human intelligence and memory. The results indicate a general belief among the sampled clinical staff that AI can facilitate clinicians' work by taking over routine tasks (e.g., distribution of medicines and dosing) and assisting with patient care (e.g., in diagnoses and examinations).

‘… artificial intelligence would be a complement, its memory capacity does not depend on the time of day or state of mind, so it complements it [the human mind] …’ Head nurse/Group 3

The results show that, according to our participants, AI will transform work by increasing, replacing and enabling tasks. For example, the number of IT professionals (who understand how AI works and affects information security) will grow with the use of AI. On the other hand, AI may decrease the work of employees such as frontline leaders or secretaries by taking over some of their duties (e.g., patient registration). The interviewees also perceived that AI could have positive effects by enabling new ways of working, potentially increasing patient contact through technology, and certainly introducing new training requirements.

‘You have to be an expert in its field to even understand what artificial intelligence can do and what possibly to do with it …’ Developer 7

### 3.2 Transforming care and services

According to the participating leaders and developers, AI will transform medical care and services by operating beside humanity. They do not believe that AI will not replace clinicians since they make the ultimate decisions concerning patient care, and AI cannot replace encounters with real humans (AI cannot replicate—or is not suitable for—human‐based interaction). In short, they did not perceive that AI will be able to replace traditional care.

‘I think AI cannot replace clinicians, it is still supplementary, because humans are still needed for interpretation’. Assistant head nurse/Group 4

The interviewees expressed perceptions that AI increases the customer orientation of services, thereby enhancing patient safety, patients' inputs in their own care, personalized care and services and their availability. It increases patient safety by decreasing the subjectivity of clinicians' interpretations, for example, when making diagnoses, and robotics may enable more precise and less invasive surgeries. It may also enable patients to become more active, for instance, ordering food automatically or staying home with the assistance of AI. AI may enable more personalized services and support preventive care. AI may also enhance services and their availability. For example, AI solutions can be used in care pathways to collect anamneses or conduct interviews. AI may also facilitate provision of services who live in remote areas.

‘… because we have robots … our surgeries can be more conservative and careful for patients …’ Nurse middle manager/Group 6

### 3.3 Transforming organizations

The interviewees expressed perceptions that AI is transforming organizations that provide specialized medical care and that its significance in specialized medical care will increase.

‘I believe that it [the role of AI] will increase once we give it a chance and sufficient resources to develop it’. Developer 3

Most leaders expressed perceptions that AI would strengthen proactive data management, for example by forecasting the kinds and numbers of patients to be expected during different months of the year.

‘Well now they are planning a program … that would collect data and enable forecasting of what kinds of patients may be expected during different months of the year’. Assistant head nurse/Group 5

The analysis also showed that participants felt that AI would enhance organizational activities, save resources and potentially increase productivity, as a much‐needed supplement.

‘… it [AI] is a much needed supplement that assists with resource savings and enhances efficiency …’ Developer 1

Finally, according to our interviewees, AI will modernize organizations' actions by changing their images and shaping internal interactions, revolutionizing organizations and their brands by making them essentially high‐tech. They also believed that interactions within organizations will become increasingly networked as AI enables different modes of communication.

‘AI and digitalization affect the organization's brand, so people have an image of a high‐tech organization’. Nurse middle manager/Group 6

## 4 DISCUSSION

We identified three main categories of content concerning nurse leaders' and digital service developers' perceptions of the future role of AI in specialized medical care: AI transforming (1) work, (2) care and services and (3) organizations. Grasping their perceptions is important for understanding the direction in which AI should be developed in specialized medical care. Our results indicate that AI may have advantages for several domains of health care, for example, patient care and both clinicians' and leaders' work. This knowledge can be utilized by developers or decision‐makers when developing or implementing new AI‐based solutions for specialized medical care. In addition, nurse leaders may utilize the findings when designing AI strategies for their organizations and developing their own AI‐related skills. Our results indicate that AI will affect organizations' brands by making them appear ‘high‐tech’ to people, which may be advantageous from a customer or employee perspective. High‐tech organizations may, for example, lure potential employees who believe that AI improves their workflows and makes work more diverse.

Although it has been suggested that AI will replace clinicians in future (Shameer et al., 2018), based on our results and earlier study this seems unlikely. In our study leaders and developers were unanimous that AI will not replace clinicians, but rather be a supportive tool and replace only routine tasks. Although it is very unlikely that AI systems will replace clinicians, then, clinicians, leaders and developers should still learn to utilize AI; indeed, Davenport and Kalakota (2019) conclude that the only clinicians who will lose their jobs because of AI are those who refuse to work alongside it. It has also been suggested that clinicians require some understanding of how AI works and how they can assess the clinical worth of its suggestions (Scott et al., 2021). Thus, we suggest that clinicians, leaders, and developers should be educated to understand and utilize AI. They should, for instance, be able to validate the so‐called black box, that is, how AI makes decisions. For leaders, understanding AI is required since they are responsible for designing organizations' AI strategies (Chen & Decary, 2020).Our results indicate that AI's role in the future will be as an assistant rather than an independent agent. This implies that AI will not be making decisions—clinicians will always be responsible for ultimate decision‐making or diagnoses. Earlier literature has also highlighted the clinician's primary role in diagnosis that may be AI‐assisted (Neri et al., 2020). Although AI enhances decision making, physicians want to understand how machine learning processes data so they can judge the trustworthiness of suggestions provided by AI (Diprose et al., 2020). According to our results, AI will facilitate and transform clinicians' work by freeing up time for patient care and transforming tasks by taking over routine work, and may provide assistance and support for clinicians in patient care. For example, the COVID‐19 pandemic markedly increased health care professionals' workloads, but some AI, such as applications developed for early COVID‐19 diagnosis, eased some of the increase (Vaishya et al., 2020). Earlier research supports our findings that AI enhances workflows and creates new roles and tasks in nursing (Buchanan et al., 2020). AI may allow more time to build relationships with patients by improving decision‐making and patient recording (Chen & Decary, 2020). In addition, robots may reduce physical workloads in patient care by assisting efforts to meet basic hygiene and care needs (Archibald & Barnard, 2018). However, there is concern that use of robots in care may affect the clinician‐patient relationship (Moyle et al., 2019), so AI should be used to enhance care rather than replace clinicians (Buchanan et al., 2020). To conclude, our results seem to be consistent with a conclusion by Chen and Decary (2020), that humans and machines have unique strengths and weaknesses, so instead of one replacing the other, they are more likely to complement each other in the optimization and provision of health care.

Our results suggest that AI will not replace traditional care and services, but it will probably increase the orientation of services towards the customer, for example by improving patient safety and enhancing personalized care. Accordingly, a previous study concluded that AI will most likely improve patient experiences, for instance by helping the identification of high‐risk population groups and assistance with health monitoring (Chen & Decary, 2020).The significance of AI seems certain to increase in future, partly because its implementation has been set as a strategic goal by organizations such as the European Commission (European Commission, 2020). The rapid development of AI also supports our finding (Yu et al., 2018). One reason to utilize more AI is the increasing amount of patient data (Chen & Decary, 2020). Nurse leaders have certainly recognized AI's potential in analysing ‘big data’, and they expect AI to strengthen proactive data management.

### 4.1 Limitations

Since all our participants worked in specialized medical care units, and most were females, the results might not be transferable to other organizations. The predominance of females in our participants can be explained by the gender distribution in the hospital: of the 140 recruited leaders only nine were male. Also, this is a single‐centre study—all the participants worked in one university hospital in Finland. Therefore, the results may not be transferable to other hospitals or countries. In addition, the transcripts were not returned to participants to comment on them or confirm their authenticity. However, during the interviews, all the participants confirmed their statements by affirming that they had nothing to add, suggesting that they had given detailed information. Finally, qualitative interview studies are inevitably subject to social desirability bias.

## 5 CONCLUSIONS

Our findings suggest that AI will have a significant role in specialized medical care. However, it will not replace clinicians or traditional services, but more likely reinforce them. Leaders and developers believe that AI‐assisted specialized medical care will have several positive consequences for work, services, and organizations. AI is still novel in health care, and there is a need to research it from different perspectives and its roles in different contexts (e.g., primary care). For example, further research from the perspective of clinicians could examine whether their experiences with AI are similar to those of leaders and developers. The experiences of patients should also be examined to develop AI‐based solutions that support services, care and truly meaningful solutions for patients.

### 5.1 Implications for nursing management

It seems inevitable that AI will plant important roles in nurse leaders' work in the future. Thus, as stated by Chen and Decary (2020), it is crucial for leaders to understand the state of AI technologies and the ways they may be used to improve service efficiency, access, and safety. Nurse leaders may also assist the establishment of organizational structures that foster positive clinicians' attitudes towards AI and provide them opportunities to be involved in all stages of AI creation (Ronquillo et al., 2021). Nurse leaders who are familiar with AI may provide better support for clinicians, management of organizations' big data, and organizational resilience. Although we suggest that nurse leaders should be familiar with the potential of AI, they should also be aware of its possible risks. Thus, we recognize needs for education and training should provide such expertise.

## CONFLICT OF INTEREST

The authors declare that they have no conflict of interests.

## ETHICS STATEMENT

Following the requirements of the research organization, no Research Ethics Committee approval was required since the interviews did not concern patients or minors, and the interviews did not intervene with physical or mental impunity (World Medical Association Declaration of Helsinki, 2013). Research permission was granted by participating organization.

## Data Availability

Research data are not shared.
